# Diversity of Lactic Acid Bacteria Involved in the Fermentation of Awa-bancha

**DOI:** 10.1264/jsme2.ME21029

**Published:** 2021-11-27

**Authors:** Hiroki Nishioka, Tomoki Ohno, Hitoshi Iwahashi, Masanori Horie

**Affiliations:** 1 Food and Biotechnology Division, Tokushima Prefectural Industrial Technology Center, 11–2 Nishibari, Saika-cho, Tokushima, Tokushima 770–8021, Japan; 2 United Graduate School of Agricultural Science, Gifu University, 1–1 Yanagido, Gifu, Gifu 501–1193, Japan; 3 The Graduate School of Natural Science and Technology, Gifu University, 1–1 Yanagido, Gifu, Gifu 501–1193, Japan; 4 Faculty of Applied Biological Sciences, Gifu University, 1–1 Yanagido, Gifu, Gifu 501–1193, Japan; 5 Health and Medical Research Institute, National Institute of Advanced Industrial Science and Technology (AIST), 2217–14 Hayashi-cho, Takamatsu, Kagawa 761–0301, Japan

**Keywords:** Awa-bancha, post-fermented tea, lactic acid bacteria, *Lactiplantibacillus pentosus*, *Lactiplantibacillus plantarum*

## Abstract

The lactic acid bacteria involved in fermentation and components in the tea leaves of Awa-bancha, a post-fermented tea produced in Naka, Kamikatsu, and Miyoshi, Tokushima, were investigated in the present study. Lactic acid bacteria were isolated from tea leaves after anaerobic fermentation and identified by multiplex PCR targeting of the *recA* gene and 16S ribosomal RNA gene homology. *Lactiplantibacillus pentosus* was the most frequently isolated species in Naka and Kamikatsu and *Lactiplantibacillus plantarum* in Miyoshi. In the phylogenetic tree based on the *dnaK* gene, *L. pentosus* isolated from Awa-bancha was roughly grouped by the production area and producer. The bacterial flora after anaerobic fermentation was dominated by *Lactiplantibacillus* spp. for most producers, and the compositions of samples from each producer varied. Organic acids, free amino acids, and catechins were analyzed as components related to the flavor of Awa-bancha. These components were unique to each producer. The present results revealed diversity in the lactic acid bacteria and flavor of Awa-bancha that depended on the producer.

Tea fermented by microorganisms is known as post-fermented tea; aerobic fermented tea is produced by fungi, anaerobic fermented tea by lactic acid bacteria, and two-stage fermented tea by both aerobic and anaerobic fermentation (Miyagawa, 1994). Post-fermented tea is only produced in a limited area of the world. In Asia, the aerobically fermented type is made in China for Pu’er tea (Guo *et al.*, 2004), while anaerobically fermented tea is produced in Thailand (Horie *et al.*, 2020) and Myanmar for Laphetso (Fukushiyama and Tain, 2009). Four types of post-fermented tea are produced in Japan. The aerobically fermented tea Batabata-cha is made in Toyama (Nakagawa, 1979; Horie *et al.*, 2019a), and the three other types come from Shikoku Island (Fig. 1). The two-stage fermented tea Ishizuchi-kurocha (Kato *et al.*, 1995; Horie *et al.*, 2019b) is produced in Saijo, Ehime, and another two-stage fermented tea, Goishi-cha (Kato *et al.*, 1994) is made in Otoyo, Kochi. Awa-bancha, the subject of the present study, is primarily produced in Naka and Kamikatsu, Tokushima. In recent years, Awa-bancha has also been made in Miyoshi, western Tokushima. Awa-bancha is an anaerobically fermented tea, and no other tea in Japan is made in a similar manner. Its production method is similar to that of Miang in Thailand (Horie *et al.*, 2020). Awa-bancha is primarily produced between July and September. The tea leaves are picked, placed directly into a kettle or basket, and boiled for a few min until the tea leaves turn brown. The boiled tea leaves are kneaded with a kneading machine and packed in a wood or plastic barrel to prevent exposure to air. The top of the barrel is then covered with a stone, and the leaves are anaerobically fermented for two to several weeks. After fermenting, the tea leaves are taken out, loosened, and dried on a rug in the sun for approximately one to three days. Nuts and branches are removed, the dried tea leaves are packed into a bag to prevent breakage, and the product is complete. The anaerobic fermentation of Awa-bancha is performed by lactic acid mainly composed of lactic acid bacteria, which play an important role. Awa-bancha has a refreshing sour taste due to the change induced in tea leaf components by anaerobic fermentation and the production of organic acids, mainly lactic acid (Kato *et al.*, 1993; Miyazaki and Nakanishi, 2007). A starter is not used, and anaerobic fermentation is performed by lactic acid bacteria in the production environment. *Lactiplantibacillus pentosus* and *Lactiplantibacillus plantarum* have been identified as the lactic acid bacteria involved in the fermentation of Awa-bancha (Okada *et al.*, 1996; Sato *et al.*, 2019; Nishioka *et al.*, 2020). Awa-bancha from each producer has a unique flavor. The diversity of lactic acid bacteria involved in fermentation may be one of the factors contributing to the individuality of Awa-bancha; however, a detailed ana­lysis that focuses on each producer has not yet been conducted. In the present study, Awa-bancha produced in Naka, Kamikatsu, and Miyoshi, Tokushima was collected for two years, and lactic acid bacteria and components related to flavor were examined.

## Materials and Methods

### Tea leaves

Awa-bancha was collected from producers in Naka, Kamikatsu, and Miyoshi, Tokushima. In 2019, samples were collected from two producers in Naka (producers A and B), two in Kamikatsu (producers C and D), and one in Miyoshi (producer E). In 2020, tea samples were collected from one producer in Naka (producer A), one in Kamikatsu (producer C), and two in Miyoshi (producers E and F). In 2020, Ishizuchi-kurocha tea leaves were collected after anaerobic fermentation from producer G in Saijo, Ehime for comparison. The tea leaves used to isolate lactic acid bacteria were stored at 4°C until used in the experiment. The tea leaves used in the component ana­lysis were freeze-dried and then powdered using a mill (IFM-700G; Iwatani).

### Isolation and identification of lactic acid bacteria from post-fermented tea

Lactic acid bacteria were isolated from tea leaves following the anaerobic fermentation of Awa-bancha and Ishizuchi-kurocha. Sterilized phosphate-buffered saline (PBS) was added to moist tea leaves after anaerobic fermentation to reach a concentration of tea leaves relative to PBS of 100‍ ‍mg mL^–1^. The mixture was then stirred with a vortex mixer for 1‍ ‍min. The supernatant was smeared on de Man, Rogosa, and Sharpe (MRS) agar plates (Becton, Dickinson and Company) and cultured in an anaerobic jar at 35°C for 2 days using AnaeroPack Kenki (Mitsubishi Gas Chemical). The colonies that grew were isolated and cultured in 10‍ ‍mL of MRS broth at 35°C for 1 day using a screw-cap test tube. After the culture broth had been centrifuged at 9,000‍ ‍rpm for 10‍ ‍min, the supernatant was removed to obtain pellets. NucleoSpin Tissue (Macherey-Nagel GmbH & Co KG) was used to extract DNA from lactic acid bacteria. A pellet was suspended in lysis solution included in the DNA extraction kit and sonicated for 5‍ ‍min using a Bioruptor (Sonic Bio). The DNA extraction operation followed the protocol included with the kit. Lactic acid bacteria species were identified by multiplex PCR targeting of the *recA* gene (Torriani *et al.*, 2001) and 16S rRNA gene homology. Multiplex PCR targeting of the *recA* gene was performed to identify members of the *L. plantarum* group (*L. plantarum*, *L. pentosus*, and *Lactiplantibacillus paraplantarum*). DNA extracted from lactic acid bacteria was used as a template. The primers used were as follows: paraF (5′-GTCACAGGCATTACGAAAAC-3′), pentF (5′-CAGTGGCGCGGTTGATATC-3′), planF (5′-CCGTTTATGCGGAACACCTA-3′), and pREV (5′-TCGGGATTACCAAACATCAC-3′). TaKaRa Ex Taq (Takara Bio) was used as the PCR enzyme. The total volume of the PCR reaction mixture was 50‍ ‍μL, and its contents were as follows: 1‍ ‍μL of template DNA, 5‍ ‍μL of 10×Ex Taq buffer (Mg^2+^-free), 1.5‍ ‍mM of MgCl_2_, 0.25‍ ‍μM each of the primers paraF, pentF, and pREV, 0.12‍ ‍μM of planF, 0.2‍ ‍mM of each deoxynucleotide triphosphate mixture, and 1.25‍ ‍U of Ex Taq polymerase. PCR amplification conditions were as follows: initial denaturation at 94°C for 3‍ ‍min, 30 cycles at 94°C for 30‍ ‍s, 56°C for 10‍ ‍s, and 72°C for 30‍ ‍s, followed by 72°C for 5‍ ‍min. The PCR amplification product was electrophoresed on a 2% agarose gel, and the appearance of bands was confirmed. The PCR amplification products of multiplex PCR targeting of the *recA* gene were 318 bp for *L. plantarum*, 218 bp for *L. pentosus*, and 107 bp for *L. paraplantarum*. Regarding lactic acid bacteria strains for which no band appeared, the 16S rRNA gene was amplified from extracted DNA with a primer for lactic acid bacteria (Lane, 1991). The primers used were 27F (5′-AGAGTTTGATCCTGGCTCAG-3′) and 1492R (5′-GGTTACCTTGTTACGACTT-3′). The total volume of the PCR reaction mixture was 50‍ ‍μL, and its contents were as follows: 1‍ ‍μL of template DNA, 5‍ ‍μL of the 10×Ex Taq buffer (Mg^2+^ plus), 1‍ ‍μM of each of the primers 27F and 1492R, 0.2‍ ‍mM of each deoxynucleotide triphosphate mixture, and 1.25‍ ‍U of Ex Taq polymerase. PCR amplification conditions were as follows: initial denaturation at 95°C for 3‍ ‍min, 40 cycles at 95°C for 30‍ ‍s, 55°C for 55‍ ‍s, and 72°C for 1‍ ‍min, followed by 72°C for 10‍ ‍min. The PCR amplification product was purified and sequenced using the Sanger method by Macrogen Japan. The sequencing primer used was LAB-SeqF (5′-TCCTGGCTCAGGACGAACGCT-3′). The sequence was searched for homology using the Standard Nucleotide BLAST of the National Center for Biotechnology Information (https://blast.ncbi.nlm.nih.gov/Blast.cgi), and the bacterial species was identified.

*L. pentosus* NBRC 106467^T^, *L. pentosus* NBRC 12011, *L. plantarum* subsp. *plantarum* NBRC15891^T^, and *L. paraplantarum* NBRC107151^T^ were purchased from the Biological Resource Center of the National Institute of Technology and Evaluation (Tokyo, Japan) for comparison with the lactic acid bacteria isolated from Awa-bancha.

### Phylogenetic analysis based on the *dnaK* gene of lactic acid bacteria

To amplify the *dnaK* gene, DNA extracted from lactic acid bacteria was used as a template with the primers Lpdnak-500F3 (5′-CCGTTCTTRTCRATRTCRAA-3′) and Lpdnak-1710R5 (5′-GAAAYYCAAGTYGGHGAAGT-3′) (Huang *et al.*, 2010). The total volume of the PCR reaction mixture was 50‍ ‍μL, and its contents were as follows: 1‍ ‍μL of template DNA, 5‍ ‍μL of the 10×Ex Taq buffer (Mg^2+^ plus), 1‍ ‍μM of each primer Lpdnak-500F3 and Lpdnak-1710R5, 0.2‍ ‍mM of each deoxynucleotide triphosphate mixture, and 1.25‍ ‍U of Ex Taq polymerase. PCR amplification conditions were as follows: initial denaturation at 94°C for 5‍ ‍min, 35 cycles at 94°C for 1‍ ‍min, 58°C for 1‍ ‍min, and 72°C for 1‍ ‍min, followed by 72°C for 7‍ ‍min. The PCR amplification product was purified and sequenced using the Sanger method by Macrogen Japan. The sequencing primer used was Lpdnak-500F3. The elucidated nucleotide sequence was aligned using ClustalW (https://clustalw.ddbj.nig.ac.jp/). The phylogenetic tree was constructed using NJplot (http://doua.prabi.fr/software/njplot). The reference strain was obtained from GenBank (https://www.ncbi.nlm.nih.gov/genbank/).

### Analysis of bacterial flora after the anaerobic fermentation of Awa-bancha

Bacterial DNA was extracted from 0.3‍ ‍g of moist tea leaves after anaerobic fermentation in 100‍ ‍μL of RNase-free water using Extrap Soil DNA Kit Plus ver.2 (Nippon Steel Eco-Tech) according to the manufacturer’ s protocol. The primers used for PCR amplification targeted the 16S rRNA gene V3–V4 region-specific portion, and 341F/805R (Klindworth *et al.*, 2013) with an Illumina adapter sequence. A 6-base index sequence for sample identification was added as the forward primer between the Illumina adapter sequence and target region-specific sequence. The target region-specific sequences used were 341F (5′-CCTACGGGNGGCWGCAG-3′) and 805R (5′-GACTACHVGGGTATCTAATCC-3′). The total volume of the PCR reaction mixture was 25‍ ‍μL, and the contents included 2.5‍ ‍μL of template DNA, 0.3‍ ‍μM each of the primers 341F and 805R, and 12.5‍ ‍μL of KAPA HiFi HS ReadyMix (KAPA Biosystems). PCR amplification conditions were as follows: initial denaturation at 95°C for 5‍ ‍min, 26 cycles at 98°C for 20‍ ‍s, 60°C for 15‍ ‍s, and 72°C for 15‍ ‍s, followed by 72°C for 5‍ ‍min. PCR amplification products were sequenced using the MiSeq system (Illumina). The resulting Fastq file was demultiplexed to each sample based on the index sequence. In this process, reads with base Q-scores less than 30 in the index part were removed. After separation, the leads were filtered by fastp (Chen *et al.*, 2018). During filtering, the primer part was removed by truncating 23 bases at the 5' end of the forward read and 21 bases at the 5' end of the reverse read. In both reads, a single base at the 3' end was truncated, reads with an average Q-score of less than 30 were removed, and low-quality bases at the 3' end (average Q-score less than 30) were truncated using a sliding window (window size 4). A sequence ana­lysis was performed on the filtered reads using QIIME 2 2021.4 (Bolyen *et al.*, 2019). DADA2 (Callahan *et al.*, 2016) (via q2-dada2) was used for sequence denoising, and amplicon sequence variants (ASVs) were created. The q2‐feature‐classifier (Bokulich *et al.*, 2018) plugin was used with the classify‐sklearn naïve Bayes taxonomy classifier for the taxonomic assignment to each ASV, and the taxonomic classifier used in this process was created based on Silva release 138.1 SSU 99% (https://www.arb-silva.de/) (Quast *et al.*, 2013; Yilmaz *et al.*, 2014) by curating with RESCRIPt (Robeson, M.S., *et al.* 2020. RESCRIPt: Reproducible sequence taxonomy reference database management for the masses. *bioRxiv* doi: https://doi.org/10.1101/2020.10.05.326504), extracting the V3–V4 region based on the amplification primer sequences, and training with the qiime feature-classifier fit-classifier-naive-bayes command. In the Curation process by RESCRIPt, the removal of low-quality sequences (containing 5 or more ambiguous bases and any homopolymers that are 8 or more bases in length), length filtering (sequences that did not meet the following criteria were removed: Archaea ≥900 bp, Bacteria ≥1,200 bp, and any Eukaryota ≥1,400 bp), and the removal of duplicate sequences were performed. ASVs with presumed chloroplast and mitochondrial origins were removed from the ASV table.

### Component ana­lysis of Awa-bancha

Organic acids were analyzed with the post-column method using an organic acid analyzer (EXTREMA; JASCO). Analytical samples were prepared by adding water to tea powder to a concentration of 100‍ ‍mg mL^–1^, shaking for 1 h, and then passing the solution through a 0.45-μm filter. An RSpak KC-811 (8×300‍ ‍mm; Showa Denko) column was used for the ana­lysis, which was conducted at 60°C. The mobile phase consisted of 3.0‍ ‍mM HClO_4_, and the flow rate was 1.0‍ ‍mL‍ ‍min^–1^. The reaction phase consisted of 0.2‍ ‍mM bromothymol blue in 15.0‍ ‍mM Na_2_HPO_4_ with a flow rate of 1.5‍ ‍mL‍ ‍min^–1^. Organic acids were detected at a wavelength of 445‍ ‍nm. Free amino acids were analyzed using a fully automatic amino acid analyzer (JLC-500V/2; JEOL). Analytical samples were prepared by adding 10% sulfosalicylic acid to tea powder to a concentration of 100‍ ‍mg‍ ‍mL^–1^, shaking for 1‍ ‍h, and then the supernatant was mixed with a lithium citrate buffer (P-21, pH‍ ‍2.98, JEOL) at a ratio of 1:1 and passed through a 0.45-μm filter. Catechins and caffeine were analyzed using high-performance liquid chromatography (Waters). Regarding samples to be analyzed, a mixture of equal parts of water and acetonitrile was added to the tea powder to a concentration of 5‍ ‍mg mL^–1^ and shaken for 40‍ ‍min, and the supernatant was then passed through a 0.45-μm filter. A Capcell Pak C18 UG120 (4.6×100‍ ‍mm; Shiseido) column was used for the ana­lysis, which was conducted at 40°C. The mobile phase consisted of 0.5% (v/v) phosphoric acid/methanol (82/18) and the flow rate was 0.8‍ ‍mL‍ ‍min^–1^. Caffeine and catechins were detected at a wavelength of 280‍ ‍nm.

## Results

### Isolation and identification of lactic acid bacteria from post-fermented tea

**Fig. 2.**
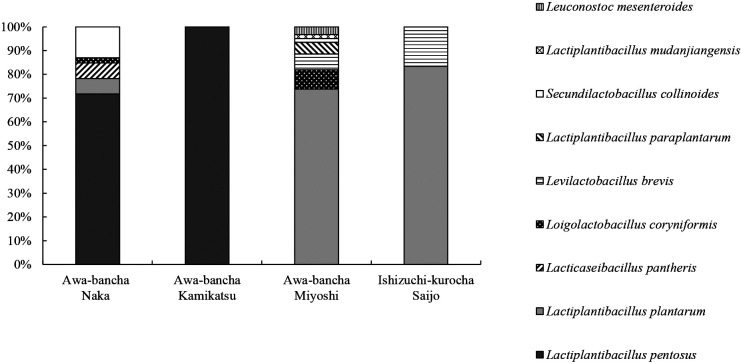
Percentage of lactic acid bacteria isolated by production area The color and pattern indicate the percentage of each lactic acid bacteria. Lactic acid bacteria were isolated using MRS agar plates from tea leaves after the anaerobic fermentation of Awa-bancha and Ishizuchi-kurocha produced in 2019 and 2020. The number of isolated Awa-bancha was 46 in Naka, 39 in Kamikatsu, 61 in Miyoshi, and 12 in Ishizuchi-kurocha.

With the use of MRS agar plates, 146 strains of lactic acid bacteria were isolated from tea leaves after the anaerobic fermentation of Awa-bancha produced in 2019 and 2020. Twelve strains of lactic acid bacteria were isolated from Ishizuchi-kurocha produced in 2020 for comparison. The species of lactic acid bacteria were identified by multiplex PCR targeting of the *recA* gene and 16S rRNA homology (Table S1). Regarding producer A in Naka, *L. pentosus* was the most frequently isolated lactic acid bacteria from two lots of Awa-bancha in 2019. Other isolates were *Secundilactobacillus collinoides*, *Lacticaseibacillus pantheris*, and *Loigolactobacillus coryniformis*. *L. pentosus* was the most frequently isolated species from the 2020 lot, and *L. plantarum* was also isolated. In the case of producer B in Naka, *L. pentosus* was the most frequently isolated species in 2019, followed by *L. plantarum*. Regarding producers C and D in Kamikatsu, only *L. pentosus* was separated in all lots. *L. plantarum* was the most frequently isolated from the two 2019 lots of producer E in Miyoshi, followed by *Levilactobacillus brevis*, *Leuconostoc mesenteroides*, and *Lactiplantibacillus mudanjiangensis*. In the 2020 lot of the same producer, *L. plantarum* was the most frequently isolated, and *L. coryniformis* and *S. collinoides* were also isolated. In the case of producer F in Miyoshi, *L. plantarum* was the most frequently isolated lactic acid bacteria, and *L. paraplantarum* and *L. coryniformis* were also isolated. Regarding Ishizuchi-kurocha by producer G in 2020, *L. plantarum* was the most frequently isolated lactic acid bacteria, followed by *L. brevis*. Fig. 2 shows the proportions of lactic acid bacteria isolated in 2019 and 2020 for each production area. Among the lactic acid bacteria isolated from Awa-bancha, *L. pentosus* was dominant in Naka and Kamikatsu, whereas *L. plantarum* was dominant in Miyoshi. *L. plantarum* was also dominant in Ishizuchi-kurocha.

### Phylogenetic ana­lysis based on the *dnaK* gene of isolated lactic acid bacteria

**Fig. 3.**
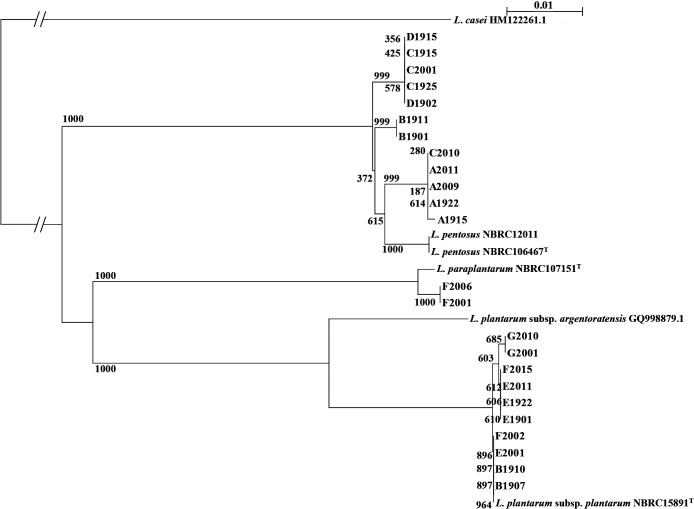
Phylogenetic tree based on the *dnaK* gene of lactic acid bacteria Numbers indicate bootstrap values calculated 1,000 times, and bars show differences in the base sequence of 1%. The sequences of* Lacticaseibacillus casei* and *Lactiplantibacillus plantarum* subsp. *argentoratensis* were obtained from GenBank. Accession numbers are shown in the figure. *L. casei* was used as the outgroup.

We analyzed *dnaK* genes in *L. pentosus*, *L. plantarum*, and *L. paraplantarum* isolated from Awa-bancha and Ishizuchi-kurocha (Table S2). We also created a phylogenetic tree based on the *dnaK* gene (Fig. 3). The lactic acid bacteria isolated from Awa-bancha were roughly grouped by the production area and producer. Furthermore, for *L. pentosus*, which was isolated from the same producer in different production years, some strains were located nearby. Similarly, *L. plantarum* isolated from Awa-bancha was also roughly grouped by the producer.

### Analysis of bacterial flora after the anaerobic fermentation of Awa-bancha

**Fig. 4.**
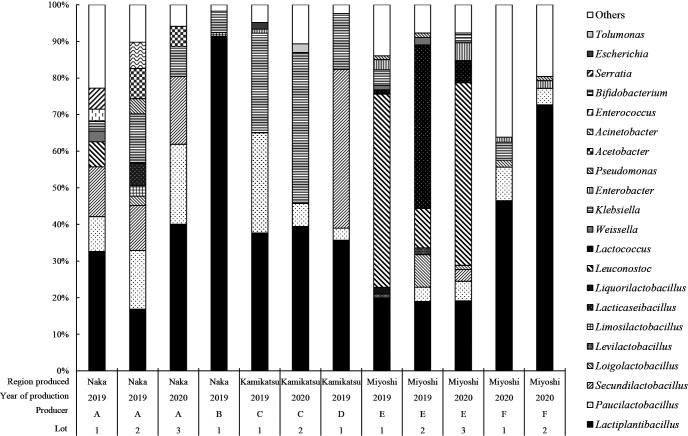
Genus-level bacterial flora of Awa-bancha after anaerobic fermentation for each producer. Colors and patterns show the proportion of each bacterium at the genus level. Bacteria with amounts less than 1% were classified as others.

The results of a bacterial flora ana­lysis of tea leaves after the anaerobic fermentation of Awa-bancha showed that *Lactiplantibacillus* spp. was the most dominant among the approximate producers, although it varied among producers (Fig. 4). *Lactiplantibacillus* spp. was detected in all producers and accounted for between 16.8 and 91.4% of the flora. Other bacteria differed among producers, and *Paucilactobacillus* spp., *Secundilactobacillus* spp. and *Klebsiella* spp. were detected. On the other hand, in the case of producer E, *Leuconostoc* spp. and* Lactococcus* spp. dominated the flora, followed by *Lactiplantibacillus* spp.

### Component ana­lysis of Awa-bancha

The components of dried tea leaves were analyzed (Table 1). An organic acid ana­lysis revealed that lactic acid was the most abundantly detected in the tea leaves of most producers after anaerobic fermentation. In addition, acetic acid and oxalic acid were detected at the second highest concentration. Acetic acid was detected at the highest concentration in lot 2 from producer A and lot 3 from producer E. The average total amount of organic acids was approximately 3,200‍ ‍mg 100 g^–1^; however, the amount for each producer differed. For example, the amount of organic acid in tea leaves from producer E was as low as approximately 900 to 1,600‍ ‍mg 100 g^–1^, while that from producer F was as high as approximately 5,400 to 5,900‍ ‍mg 100 g^–1^. In the ana­lysis of catechins, EGC was the most abundant for most producers, followed by EGCg. The average total amount of catechins was approximately 6,700‍ ‍mg 100 g^–1^; however, this also varied among producers. Producer A had a small amount of catechins, approximately 1,500 to 3,900‍ ‍mg 100‍ ‍g^–1^, producer C had a large amount, approximately 9,200 to 10,700‍ ‍mg 100 g^–1^, and producer F had a large amount approximately 11,700 to 12,600‍ ‍mg 100 g^–1^. In the free amino acid ana­lysis, theanine was detected at the highest amount, followed by glutamic acid. In addition, a large amount of γ-aminobutyric acid was detected in lot 2 of producer A and in lots 2 and 3 of producer E.

## Discussion

In the present study, the lactic acid bacteria involved in the fermentation of Awa-bancha and the components related to flavor were investigated for two years. The lactic acid bacteria involved in Awa-bancha fermentation were diverse and depended on the production area and producer. The isolation of lactic acid bacteria on MRS agar plates revealed that *L. pentosus* was the most frequently isolated among the producers (A, B, C, and D) in Naka and Kamikatsu. On the other hand, *L. plantarum* was the most frequently isolated among the producers (E and F) in Miyoshi. In addition, the dominant lactic acid bacteria were the same, even in different production years. Geographically, Naka and Kamikatsu are adjacent to each other, while Miyoshi is approximately 70‍ ‍km west, with Mt. Tsurugi, at an altitude of 1,955‍ ‍m, between them. *L. pentosus* and *L. plantarum* are classified as the *L. plantarum* phylogenetic group (Hirayama and Endo, 2016) and have been isolated from various plant environments (Okada, 2002; Parente *et al.*, 2010). These genotypes are very close, with more than 99% homology in 16S rRNA (Torriani *et al.*, 2001; Bringel *et al.*, 2005). Therefore, *L. pentosus* and *L. plantarum* are very close species; however, it currently remains unknown why the dominant species involved in the fermentation of Awa-bancha differs depending on the production area. *L. plantarum* was the most frequently isolated from producer G of Ishizuchi-kurocha in Saijo, and similar results were obtained for producers E and F in Miyoshi. The lactic acid bacteria in each region are presumed to be different; however, further clarification is expected in the future. The phylogenetic tree based on the *dnaK* gene suggested that unique lactic acid bacteria were established in each producer at the strain level. The genome sizes of *L. pentosus* and *L. plantarum* are larger than 3‍ ‍Mb (Kleerebezem *et al.*, 2003; Huang *et al.*, 2018; Niwa *et al.*, 2020); therefore, the properties of each strain and metabolites may markedly differ, thereby affecting the flavor of Awa-bancha. Lactic acid bacterial isolates other than *L. pentosus* and *L. plantarum* also differed from producer to producer. In the case of producer A, *S. collinoides*, *L. pantheris*, and *L. coryniformis* were isolated in addition to *L. pentosus*, and previous studies reported the isolation of the same bacteria from Awa-bancha (Sato *et al.*, 2019; Nishioka *et al.*, 2020). *L. pantheris* was previously isolated from Ishizuchi-kurocha and Miang and *L. coryniformis* from Miang (Chaikaew *et al.*, 2017; Horie *et al.*, 2019b). In the samples of producer B, *L. pentosus* was the most frequently isolated, followed by *L. plantarum*. Only *L. pentosus* was isolated from producers C and D. In the case of the tea of producer E, *L. brevis*, *L. coryniformis*, *S. collinoides*, *L. mesenteroides*, and *L. mudanjiangensis* were isolated in addition to *L. plantarum*. *L. brevis* is frequently isolated from Ishizuchi-kurocha and occasionally from Awa-bancha (Horie *et al.*, 2019b; Nishioka *et al.*, 2020). *L. mesenteroides* has been isolated from Awa-bancha and Miang (Chaikaew *et al.*, 2017; Sato *et al.*, 2019). *L. mudanjiangensis* was not previously isolated from Awa-bancha or other post-fermented teas and was identified for the first time in the present study. *L. paraplantarum*, which has not previously been isolated from Awa-bancha or other post-fermented teas, was isolated from producer F.

*Lactiplantibacillus* spp. was dominant in the bacterial flora ana­lysis of tea from most producers. A previous study reported that the microorganisms involved in the fermentation of Awa-bancha were selected by the polyphenols contained in tea leaves as a control factor (Uchino *et al.*, 2020). The bacterial flora differed for each producer. *Acetobacter* spp. were more abundant in lots 2 and 3 of producer A than in the samples from other producers. Producer C has a large proportion of *Klebsiella* spp. The results from producer E were dominated by *Leuconostoc* spp. and *Lactococcus* spp., but not *Lactiplantibacillus* spp. The components of the leaves after anaerobic fermentation were similar among producers. Lactic acid was detected by an organic acid ana­lysis in the samples of most producers. Lactic acid is produced by lactic acid bacteria, such as *Lactiplantibacillus* spp. On the other hand, in lot 2 of producer A, more acetic acid was detected than lactic acid, which may have been due to the bacterial flora ana­lysis of lot 2 from producer A detecting more *Acetobacter* spp. than in the samples from the other producers. Since *Acetobacter* spp. is an aerobic bacterium, air may have been present during fermentation. Therefore, organic acids, such as lactic acid and acetic acid, are expected to produce the acidity in Awa-bancha. The ana­lysis of catechins showed that EGC was the most abundant in the tea from many producers, followed by EGCg. Catechins contribute to the astringent taste of Awa-bancha and also exert antioxidant effects (Hara, 2000). EGCg in tea leaves was previously shown to be hydrolyzed to EGC and gallic acid by lactic acid bacteria with tannase activity (Osawa *et al.*, 2000). Free catechins have also been reported to be less astringent than gallate catechins and are efficiently absorbed into the intestinal tract (Henning *et al.*, 2005). An ana­lysis of free amino acids detected large amounts of theanine and glutamic acid. Theanine enhances the flavor of other umami components (Narukawa *et al.*, 2008) and has also been reported to improve sleep and relaxation (Kobayashi *et al.*, 1998; Ozeki *et al.*, 2004). Glutamic acid is regarded as an umami component of tea. In addition, more γ-aminobutyric acid was detected in the tea of producer E than in that of the other producers. This component has health benefits, such as promoting relaxation (Abdou *et al.*, 2006), which may be related to the isolation of *L. brevis*, a high producer of γ-aminobutyric acid, from the samples of producer E. (Yokoyama *et al.*, 2002; Mori *et al.*, 2007). In the present study, the lactic acid bacteria involved in the fermentation of Awa-bancha were dominated by *Lactiplantibacillus* spp., mainly *L. pentosus* and *L. plantarum*, among most producers. The components produced by these lactic acid bacteria were considered to significantly contribute to the flavor of Awa-bancha. The quality of Awa-bancha is improved by preparing an optimal environment for lactic acid bacteria. For example, it is important to prevent air from entering during anaerobic fermentation. Therefore, lactic acid bacteria appear to affect the components of Awa-bancha; however, there were also contradictions. For example, *L. brevis* was not isolated from lots 1 and 2 of producer A, whereas γ-aminobutyric acid was abundant. In addition, in the bacterial flora after anaerobic fermentation, producer B had a higher proportion of *Lactiplantibacillus* spp. than other producers, whereas the amount of lactic acid and total amount of organic acid were not high. Collectively, the present results suggest diversity in the lactic acid bacteria and components of Awa-bancha among different producers; however, the relationship between them remains unclear. Further studies are warranted to elucidate the mechanisms by which lactic acid bacteria and production conditions affect the components of Awa-bancha.

## Citation

Nishioka, H., Ohno, T., Iwahashi, H., and Horie, M. (2021) Diversity of Lactic Acid Bacteria Involved in the Fermentation of Awa-bancha. *Microbes Environ ***36**: ME21029.

https://doi.org/10.1264/jsme2.ME21029

## Supplementary Material

Supplementary Material

## Figures and Tables

**Fig. 1.**
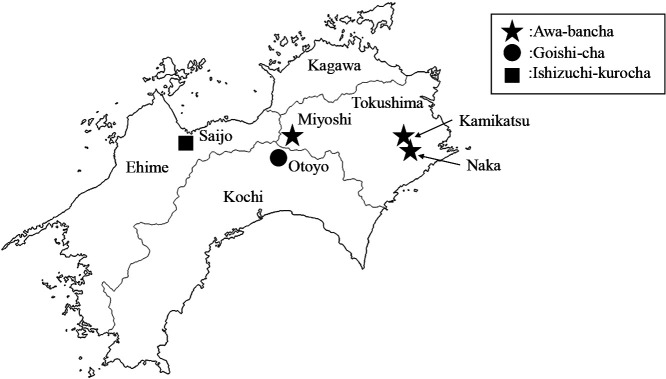
Production area of post-fermented tea in Shikoku Island

**Table 1.** Components of Awa-bancha. mg 100 g^–1^

Region produced		Naka	Naka	Naka	Naka	Kamikatsu	Kamikatsu	Kamikatsu	Miyoshi	Miyoshi	Miyoshi	Miyoshi	Miyoshi
Year of production		2019	2019	2020	2019	2019	2020	2019	2019	2019	2020	2020	2020
Producer		A	A	A	B	C	C	D	E	E	E	F	F
Lot		1	2	3	1	1	2	1	1	2	3	1	2
Organic acids	Oxalic acid	126.1	233.2	732.4	183.2	372.7	709.8	669.9	130.9	112.2	358.8	2,260.7	1,506.4
	Citric acid	13.9	—	80.7	76.4	114.3	235.1	367.1	—	—	17.4	59.1	69.1
	Malic acid	—	—	36.7	—	—	—	16.2	—	—	24.3	26.9	29.0
	Succinic acid	280.5	126.0	226.0	206.1	195.1	286.0	287.5	90.3	113.5	236.8	519.9	427.9
	Lactic acid	3,505.5	688.8	2,399.3	2,297.6	829.4	1,618.6	1,237.1	561.1	958.3	83.4	2,751.2	3,036.5
	Acetic acid	623.7	1,773.1	817.5	382.6	385.5	489.4	849.3	133.4	352.7	891.6	267.0	342.6
	Total organic acids	4,549.7	2,821.1	4,292.6	3,145.9	1,897.1	3,338.9	3,427.0	915.8	1,536.7	1,612.3	5,884.8	5,411.3
Catechins	EGC	1,123.0	1,081.4	1,893.4	2,013.3	5,528.9	6,145.4	2,531.5	2,315.6	2,942.8	5,441.4	6,685.7	6,656.2
	C	371.0	312.5	403.3	57.9	161.1	203.7	37.4	149.1	164.7	—	289.6	249.9
	EGCg	1,001.3	105.4	1,231.2	3,084.5	1,625.0	2,192.8	960.6	3,484.4	1,921.2	803.6	3,738.3	3,032.8
	EC	111.6	—	—	91.8	1,402.6	1,401.8	—	101.6	—	—	1,265.9	1,159.7
	ECg	247.0	—	340.2	644.3	477.9	772.7	322.9	601.8	353.1	156.6	603.1	553.7
	Total catechins	2,853.8	1,499.3	3,868.2	5,891.8	9,195.5	10,716.3	3,852.4	6,652.5	5,381.8	6,401.6	12,582.6	11,652.3
Caffeine		591.2	1,744.2	1,453.4	497.5	1,392.5	729.6	1,004.0	1,652.1	1,368.6	1,485.4	1,541.3	466.8
Free amino acids	O-Phosphoserine	12.9	22.9	16.6	13.0	12.1	13.2	17.4	7.2	9.3	10.5	11.5	6.7
	Taurine	5.5	24.7	8.7	7.1	3.2	3.1	—	4.1	6.2	4.2	3.4	2.2
	O-Phosphoethanolamine	2.4	4.4	2.1	3.3	2.7	3.2	2.5	1.8	1.5	1.3	3.0	—
	Threonine	0.5	0.8	—	—	0.7	—	0.5	—	—	—	—	—
	Serine	1.4	2.1	0.8	1.2	1.6	0.8	2.1	1.8	0.7	3.1	2.0	1.3
	Asparagine	—	8.0	—	12.5	—	—	—	11.9	16.9	—	—	—
	Glutamic acid	90.2	44.8	27.8	112.0	80.0	118.4	115.0	67.5	14.7	19.7	97.3	44.6
	Glutamine	—	—	3.1	—	1.1	—	—	—	—	—	—	2.5
	Theanine	187.9	99.6	145.8	338.2	122.2	18.7	187.2	298.8	468.5	404.2	148.6	83.1
	Glycine	2.1	12.1	3.3	3.5	1.3	2.0	0.4	11.5	2.6	0.8	4.0	1.8
	Alanine	11.8	96.1	22.3	33.7	15.5	18.7	19.8	0.0	16.2	8.3	21.8	16.7
	Citrulline	2.6	—	—	—	—	—	—	—	—	—	—	—
	α-Aminobutyric acid	1.1	2.6	2.8	1.1	2.1	2.1	19.8	—	1.1	3.5	3.9	2.0
	Valine	4.1	4.7	3.5	11.0	3.1	2.0	8.4	8.5	6.6	7.1	4.6	2.5
	Cystine	—	—	—	—	1.4	—	—	—	—	—	—	—
	Cystathionine	—	2.0	—	0.2	—	—	0.2	—	—	—	—	—
	Isoleucine	—	5.3	—	—	—	—	—	—	—	—	—	—
	Leucine	—	19.1	0.4	1.1	—	—	0.3	—	0.3	—	0.3	—
	Tyrosine	—	8.8	—	—	—	—	—	—	—	—	—	—
	Phenylalanine	—	11.0	—	2.4	0.7	—	—	—	—	—	—	—
	γ-Aminobutyric acid	25.7	80.5	11.4	10.7	15.0	7.2	2.2	22.1	65.6	38.3	4.5	2.3
	2-Aminoethanol	—	—	—	—	—	—	—	—	—	—	—	—
	Ammonium chloride	5.9	30.9	7.6	13.0	11.1	8.6	11.9	13.0	17.2	17.3	25.5	16.6
	5-Hydroxylysine	—	—	—	—	—	—	—	—	—	—	—	—
	Ornithine	31.3	2.1	19.9	1.7	—	—	9.8	3.7	1.4	2.6	—	—
	1-Methyl-L-histidine	—	—	—	—	0.3	—	—	—	—	—	—	—
	Histidine	—	—	—	2.0	1.2	—	—	—	0.4	—	—	—
	Lysine	1.2	1.2	5.1	2.4	0.3	—	—	0.6	1.6	—	3.2	2.8
	Tryptophan	—	7.2	—	6.9	8.8	—	—	—	—	—	—	—
	Arginine	—	—	—	4.2	7.0	—	—	—	—	—	—	—
	Hydroxyproline	26.8	6.0	8.5	21.1	8.2	7.4	10.4	22.3	37.4	14.3	7.8	4.0
	Proline	3.9	2.4	4.8	—	3.0	1.6	3.0	—	—	1.6	3.3	1.7
	Total free amino acids	417.4	499.2	294.4	602.2	302.8	207.0	411.0	474.9	668.3	536.9	344.7	190.9

—: Overlapped with other peaks or below the detectable level. EGC: Epigallocatechin, C: Catechin, EGCg: Epigallocatechin gallate, EC: Epicatechin, ECg: Epicatechin gallate. The following components were not detected: Pyroglutamic acid, Urea, Aspartic acid, 2-Aminoadipic acid, Methionine, β-Alanine, β-Aminoisobutyric acid, Anserine, Carnosine, and 3-Methyl-L-histidine.
