# Supplementary material for: Diversity of Lactic Acid Bacteria Involved in the Fermentation of Awa-bancha
Source: Microbes Environ. 2021 Nov 27;36(4):ME21029. doi: 10.1264/jsme2.ME21029 (PMC8674441; doi:10.1264/jsme2.ME21029)
Supplement: Supplementary file 1 — Supplementary Material [file 36_21029_s1.pdf]

Table S1. Number of lactic acid bacteria isolated from Awa-bancha and Ishizuchi-kurocha using MRS agar plates for each producer.

Types of post-fermented tea	Awa-bancha	Awa-bancha	Awa-bancha	Awa-bancha	Awa-bancha	Awa-bancha	Awa-bancha	Awa-bancha	Awa-bancha	Awa-bancha	Awa-bancha	Awa-bancha	Ishizuchi-kurocha
Region produced	Naka	Naka	Naka	Naka	Kamikatsu	Kamikatsu	Kamikatsu	Miyoshi	Miyoshi	Miyoshi	Miyoshi	Miyoshi	Saijo
Year of produce	2019	2019	2020	2019	2019	2020	2019	2019	2019	2020	2020	2020	2020
Producer	A	A	A	B	C	C	D	E	E	E	F	F	G
Lot	1	2	3	1	1	2	1	1	2	3	1	2	1
Lactiplantibacillus pentosus	8	8	11	6	12	12	15	-	-	-	-	-	-
Lactiplantibacillus plantarum	-	-	1	2	-	-	-	16	6	7	7	9	10
Lactiplantibacillus paraplantarum	-	-	-	-	-	-	-	-	-	-	3	-	-
Secundilactobacillus collinoides	3	3	-	-	-	-	-	-	-	1	-	-	-
Lacticaseibacillus pantheris	-	3	-	-	-	-	-	-	-	-	-	-	-
Loigolactobacillus coryniformis	-	1	-	-	-	-	-	-	-	3	-	2	-
Levilactobacillus brevis	-	-	-	-	-	-	-	3	1	-	-	-	2
Lactiplantibacillus mudanjiangensis	-	-	-	-	-	-	-	-	1	-	-	-	-
Leuconostoc mesenteroides	-	-	-	-	-	-	-	2	-	-	-	-	-
Total isolates	11	15	12	8	12	12	15	21	8	11	10	11	12

- : Not isolated

Table S2. Lactic acid bacteria used in the phylogenetic analysis of the *dnaK* gene.

Strain	Species	DDBJ Accession No.	Isolation				
			Source	Region	Year	Producer	Lot
NBRC 106467[T]	*Lactiplantibacillus pentosus*	LC625743	Corn silage				
NBRC 12011	*Lactiplantibacillus pentosus*	LC625744					
NBRC 15891[T]	*Lactiplantibacillus plantarum*	LC625745	Pickled cabbage				
NBRC 107151[T]	*Lactiplantibacillus paraplantarum*	LC625770	Beer spoilage agent				
A1915	*Lactiplantibacillus pentosus*	LC625746	Awa-bancha	Naka	2019	A	1
A1922	*Lactiplantibacillus pentosus*	LC625747	Awa-bancha	Naka	2019	A	2
A2009	*Lactiplantibacillus pentosus*	LC625748	Awa-bancha	Naka	2020	A	3
A2011	*Lactiplantibacillus pentosus*	LC625749	Awa-bancha	Naka	2020	A	3
B1901	*Lactiplantibacillus pentosus*	LC625750	Awa-bancha	Naka	2019	B	1
B1907	*Lactiplantibacillus plantarum*	LC625751	Awa-bancha	Naka	2019	B	1
B1910	*Lactiplantibacillus plantarum*	LC625752	Awa-bancha	Naka	2019	B	1
B1911	*Lactiplantibacillus pentosus*	LC625753	Awa-bancha	Naka	2019	B	1
C1915	*Lactiplantibacillus pentosus*	LC625754	Awa-bancha	Kamikatsu	2019	C	1
C1925	*Lactiplantibacillus pentosus*	LC625755	Awa-bancha	Kamikatsu	2019	C	1
C2001	*Lactiplantibacillus pentosus*	LC625756	Awa-bancha	Kamikatsu	2020	C	2
C2010	*Lactiplantibacillus pentosus*	LC625757	Awa-bancha	Kamikatsu	2020	C	2
D1902	*Lactiplantibacillus pentosus*	LC625758	Awa-bancha	Kamikatsu	2019	D	1
D1915	*Lactiplantibacillus pentosus*	LC625759	Awa-bancha	Kamikatsu	2019	D	1
E1901	*Lactiplantibacillus plantarum*	LC625760	Awa-bancha	Miyoshi	2019	E	1
E1922	*Lactiplantibacillus plantarum*	LC625761	Awa-bancha	Miyoshi	2019	E	2
E2001	*Lactiplantibacillus plantarum*	LC625762	Awa-bancha	Miyoshi	2020	E	3
E2011	*Lactiplantibacillus plantarum*	LC625763	Awa-bancha	Miyoshi	2020	E	3
F2001	*Lactiplantibacillus paraplantarum*	LC625764	Awa-bancha	Miyoshi	2020	F	1
F2002	*Lactiplantibacillus plantarum*	LC625765	Awa-bancha	Miyoshi	2020	F	1
F2006	*Lactiplantibacillus paraplantarum*	LC625766	Awa-bancha	Miyoshi	2020	F	1
F2015	*Lactiplantibacillus plantarum*	LC625767	Awa-bancha	Miyoshi	2020	F	2
G2001	*Lactiplantibacillus plantarum*	LC625768	Ishizuchi-kurocha	Saijo	2020	G	1
G2010	*Lactiplantibacillus plantarum*	LC625769	Ishizuchi-kurocha	Saijo	2020	G	1
